# Trajectories of Posttraumatic Growth and Their Associations With Quality of Life After the 2011 Tohoku Earthquake and Tsunami

**DOI:** 10.1002/jts.22628

**Published:** 2020-11-23

**Authors:** Yasushi Kyutoku, Ippeita Dan, Mitsuru Yamashina, Ren Komiyama, Angela J. Liegey‐Dougall

**Affiliations:** ^1^ Research and Development Initiative Chuo University Tokyo Japan; ^2^ Department of Integrated Science and Engineering for Sustainable Societies, Faculty of Science and Engineering Chuo University Tokyo Japan; ^3^ Department of Psychology, Faculty of Letters Chuo University Tokyo Japan; ^4^ Department of Psychology College of Science University of Texas at Arlington Arlington Texas USA

## Abstract

The 2011 Tohoku earthquake and tsunami in Japan was an extraordinarily stressful incident that caused harmful psychological reactions, such as posttraumatic stress disorder (PTSD), among affected individuals. However, a proportion of exposed individuals experienced posttraumatic growth (PTG), characterized by a noticeable degree of personal strength, spirituality, life appreciation, perception of new possibilities in life, and enhanced relationships with others. Some researchers have argued that these positive reactions may be an illusory change related to coping with traumatic events. We examined trajectory patterns related to PTG Inventory (PTGI) subscales to elucidate the existence of both real and illusory growth regarding quality of life (QoL), utilizing group‐based trajectory models. Three online questionnaires were distributed at 6 months (*N* = 2,554; *M* age = 47.04 years, *SD* = 12.62), 12 months (*N* = 887; *M* age = 48.11 years, *SD* = 12.43), and 42 months (*N* = 560; *M* age = 48.86 years, *SD* = 12.25) postdisaster. Participants responded to items related to demographic characteristics, disaster experiences, posttraumatic stress symptoms, PTG, and QoL. Three main PTG trajectories emerged, characterized by growth, no growth, and illusory growth, with QoL as a time invariant covariate. Compared with the growth trajectory, the odds ratios (*OR*s) for no growth ranged from 2.27 to 5.04; for illusory growth, the *OR*s ranged from 2.09 to 4.67. To our knowledge, this was the first study to report growth trajectories related to PTGI subscales and their underlying differences in psychological mechanisms and processes following the 2011 Tohoku earthquake and tsunami.

The Tohoku Earthquake, which took place on March 11, 2011, was the most powerful earthquake ever recorded in Japan, registering a magnitude of 9.0 on the Richter scale. The subsequent tsunami inflicted severe damage on the Pacific coast of northeastern Japan. Further, these natural disasters led to a technological disaster, the Fukushima I Nuclear Power Plant disaster. Due to the disaster's complexity, the cumulative number of earthquake, tsunami, and power plant victims included 18,131 fatalities and 2,829 missing persons (Fire and Disaster Management Agency of Japan, 2018). As the incident was extraordinarily stressful, it was considered to be a potentially traumatic event (PTE) for individuals both directly and indirectly exposed to the damage it caused (Galea et al., 2005; Katz et al., 2002; Nemeroff et al., 2006; Neria et al., 2008; Norris, Friedman, & Watson, 2002; Norris, Friedman, Watson, et al., 2002; Norris et al., 2006). It is important to note that PTEs can elicit not only harmful psychological reactions but also potentially beneficial reactions (Calhoun & Tedeschi, 1989, 2006, 2014; Dekel et al., 2012; Hall et al., 2010; Lechner et al., 2009; Neria et al., 2008; Tedeschi & Calhoun, 1996). Posttraumatic stress symptoms (PTSS) are one of the most frequently reported insalubrious psychological reactions following a PTE (Lechner et al., 2009; Norris, Friedman, & Watson, 2002). Posttraumatic stress disorder (PTSD) symptoms include intrusive recollections and avoidance of the event, heightened negative thoughts and feelings, and event‐related arousal and reactivity that lasts longer than 1 month. These symptoms may persist and become severe, often leading to a diagnosis of PTSD (American Psychiatric Association [APA], 2000). The prevalence rates of PTSD following a complex disaster associated with an earthquake have been reported to be between 8.6% and 49.6% (Armenian et al., 2000; Cao et al., 2003; Kun et al., 2009; Liu et al., 2006; McMillen et al., 2000). The initial level of and prognosis regarding PTSS depend largely on victim‐level characteristics, the severity of subjective and objective exposure to the source of the trauma, and the type of PTE experienced (Neria et al., 2008).

On the other hand, a potentially beneficial post‐PTE psychological reaction, known as posttraumatic growth (PTG; Tedeschi & Calhoun, 1996), has also been reported. Posttraumatic growth has been defined as “[transformative psychological growth] anchored in distress that reaches far beyond everyday stressors” (Werdel & Wicks, 2012). Through the struggles associated with PTE exposure, a certain proportion of individuals experience a noticeable degree of refined relationships with others, an enhanced level of personal strength, changes in life priorities, a stronger sense of spirituality, a greater appreciation of life, and the perception of new possibilities (Tedeschi & Calhoun, 1996). Various types of PTEs, including technological disasters, natural disasters, man‐made disasters, illness, injury, and loss of loved ones, have been reported to foster PTG (Helgeson et al., 2006).

However, the course of PTG following a PTE has not been adequately studied due to the cross‐sectional nature of most investigations. Recent studies have suggested that there may be multiple trajectories underlying the course of PTG following a PTE. In a nationally representative sample of U.S. veterans, two assessments of PTG conducted approximately two years apart identified five distinct PTG trajectories (Tsai et al., 2016), given here in the order of prevalence found in the sample: low PTG, moderately declining PTG, increasing PTG, dramatically declining PTG, and consistent PTG. However, in a subsequent 4‐year follow‐up, only three PTG trajectories were identified: low and decreasing PTG, moderate PTG, and high and increasing PTG (Tsai & Pietrzak, 2017). Recent studies among cancer patients have identified four to six PTG trajectories across four assessments during 12‐ to 24‐month time spans (e.g., Danhauer et al., 2015; Husson et al., 2017; Pat‐Horenczyk et al., 2016; Wang et al., 2014). Regarding PTG following the 2008 Wenchuan earthquake in China, four trajectories, including high PTG, increasing PTG, low PTG, and decreasing PTG trajectories, were found (Zhou et al., 2019). In summary, the literature reports four trajectories or a combination of those trajectories that have been recurrently extracted, representing high levels of PTG, low levels of PTG, decreasing PTG, and increasing PTG. These findings indicate the importance of further delineating PTG trajectories after a PTE, understanding their meaning, and determining how these trajectories are associated with long‐term health outcomes.

Posttraumatic growth has often been conceptualized as the emergence of multifaceted positive benefits following a PTE (Taku et al., 2008; Tedeschi & Calhoun, 1996). Subcomponents of PTG have included relationships with others, new possibilities, personal strength, spiritual change, and appreciation of life. The multifaceted nature of PTG has induced some researchers to argue that these positive sequelae may not be real. Frazier et al. (2009) argued that positive change in the aftermath of a PTE may be misperceived coping. Others, such as Zoellner and Maecker (2006), have advocated the “Janus face” model of PTG to resolve disagreements between the actual and illusory PTG models. According to this model, PTG has both a thriving face and an illusory face, which is not necessarily good or bad. Rather, it is a transiently distorted perception of growth to buffer the effects of a stressful event during a relatively early posttraumatic period. The model posits that illusory growth decreases with time if one does not experience actual PTG, whereas actual PTG increases or remains stably high with time. Pat‐Horenczyk et al. (2016) defined stable PTG as a constructive PTG trajectory and decreasing PTG as an illusory PTG trajectory. These terminological, conceptual, empirical, and temporal differences in PTG may be reflected in multiple trajectories. This leads to the primary aim of the current study: To examine patterns of PTG change over time by elucidating different PTG trajectories. More specifically, based on these differences in conceptual frameworks, we operationalized the following trajectory patterns. First, we posited that individuals who faced a PTE would show very low levels of PTG or no PTG over time if they did not perceive growth; we operationally named this the “no PTG” trajectory. Second, we hypothesized that individuals display a decreasing trajectory if their perceived growth was illusory only during the early posttraumatic phase, as described in Zoellner and Maercker's model (2006) and previous findings (Husson et al., 2017; Tsai et al., 2016, 2017). We named this trajectory “illusory PTG.” Third, we expected some participants to show late‐onset PTG, which has been demonstrated in previous research (Johannesson et al., 2015). We operationally named this trajectory “late‐onset PTG.” Fourth, we posited that individuals who experienced transformative growth would show constantly high or moderately high levels of PTG over time (e.g., Pat‐Horenczyk et al., 2016; Tedeschi & Calhoun, 1996). We operationally named this trajectory “PTG.” Thus, we expected to extract four distinct trajectories: no PTG, illusory PTG, late‐onset PTG, and PTG. Further, we hypothesized that each facet of PTG may have a different trajectory path during an individual's period of psychological adjustment. However, trajectories for different facets of PTG have not yet been elucidated. Therefore, we aimed to examine the hypothesized PTG trajectory for each facet.

In addition, because improvements in quality of life (QoL) have been regarded as a general indicator of psychological adjustment (Teodorescu et al., 2012), the current study focused on QoL as an adjustment outcome. The association between PTG and QoL has not been straightforward in the previous literature. Some studies have reported a positive association (Teodorescu et al., 2012), whereas others have reported an inverse association or no association (Bellizzi et al., 2010; Linley & Joseph, 2004). The existence of multiple PTG trajectories may explain these mixed findings. Therefore, our second study aim was to examine whether there was an association between PTG trajectory and QoL. Based on our previous findings (Kyutoku et al., 2012), we expected PTG related to the 2011 Tohoku Earthquake and Tsunami to be positively associated with QoL. Thus, we hypothesized that constructive PTG (i.e., PTG trajectory) would be more positively associated with subsequent QoL than PTG trajectories that were not considered to be constructive (i.e., no PTG and illusory PTG trajectories). The absence of differences in QoL ratings between individuals categorized in the no PTG trajectory group and those in the PTG trajectory group would indicate that the no PTG trajectory group included individuals who were resilient enough to have not experienced PTSS following the disaster (Bonnano, 2005). In summary, the current study was conducted first to examine patterns of PTG change at 6 months, 12 months, and 42 months following the 2011 Tohoku earthquake, tsunami, and power plant disaster as well as to examine the association between the illusory PTG trajectory and QoL at 42 months.

## Method

### Participants and Procedure

Participant characteristics are presented in Table 1. A survey company (Cross Marketing Inc., Tokyo, Japan; *N*_participant pool_ > 1.8 million at the time of measurement) was contracted to collect online responses. The current study used measurements conducted 6, 12, and 42 months after the disaster. Three months prior to the first measurement (i.e., June 2011), a screening study was conducted for ethical reasons to exclude participants with excessively high PTSS levels. The screening study, part of the study reported in Kyutoku et al. (2012), was used exclusively for screening purposes for the current manuscript. In total, 6,043 individuals were randomly invited to participate in the screening study, and those who voluntarily agreed to participate were asked to complete the online survey. After agreeing to the study terms, participants responded to questions regarding age and gender and completed the Japanese version of the Impact of Events Scale–Revised (IES‐R). Participants who scored higher than 61 out of 88 on the IES‐R, using the Tohoku earthquake and tsunami (Kyutoku et al., 2012) and the 1995 Great Hanshin earthquake (Asukai et al., 2002) as focal PTEs, were withdrawn from the study due to an agreement with the institutional ethical committee to prevent the aggravation of possible PTSD and were given the recommendation to consult with the public health care department along with relevant contact information. Note that PTS scores obtained at this assessment point were used solely for screening purposes and were not included in the following analyses. In total, 18 potential participants did not meet the PTS criteria (*M* age = 44.94 years, *SD* = 13.10; *n* = 9 men, *n* = 9 women), including 15 from the primary victimized area (described later), and data collection was stopped for these individuals. Individuals who met the PTS criteria (*N* = 3,455) proceeded to questions for the main study regarding demographic information, self‐reported health conditions, and experiences with the disaster.

**Table 1:** Participant Characteristics

	6 months		12 months		42 months	
Variable	*n*	%	*n*	%	*n*	%
Gender						
Men	1,491	58.4	536	60.4	360	64.3
Women	1,063	41.6	351	39.6	200	35.7
Marital status						
Married	1,692	66.1	607	68.4	375	67.0
Unmarried	757	30.9	280	31.6	185	33.0
Parents of children	1,540	60.3	526	59.3	336	60.0
Education						
Junior high school	53	2.1	26	2.9	15	2.7
High school	750	29.4	285	32.2	170	30.4
Associate's degree	559	21.9	181	20.5	115	20.5
College degree	1,068	41.8	361	40.8	235	42.1
Graduate school	112	4.4	31	3.5	23	4.1
Household income (JPY)^a^						
< ¥3,990,000	664	26.0	220	28.2	132	26.3
¥4,000,000–¥5,999,000	634	24.8	215	27.6	136	27.2
¥6,000,000–¥7,999,999	403	15.8	144	18.0	103	20.6
¥8,000,000–¥9,999,999	290	11.4	115	14.8	74	14.8
≥ ¥10,000,000	267	10.4	86	11.1	56	11.2
Employment status						
Full time	1,416	54.4	478	53.9	383	68.4
Part time	276	10.8	84	9.5	52	9.3
Student	53	2.1	9	1.0	5	0.9
Unemployed	306	12.0	141	15.9	91	16.3
Housewife/husband	457	17.9	158	17.8	86	15.4
Other	46	1.8	17	1.9	13	2.3
Self‐reported health status						
Psychosomatic complaints	571	22.4	204	23.0	128	22.9
Mental disorder	129	5.1	44	5.0	32	5.7
Medication	591	23.1	208	23.4	136	24.3
Drinker of alcohol	1,121	43.9	409	46.1	264	47.1
Smoker	608	23.8	219	24.7	145	25.9
Disaster‐related experiences						
Financial loss	815	31.9	291	32.8	187	33.4
Acquaintance victimized	1,001	39.2	359	40.5	220	39.3
Acquaintance's death	409	16.0	144	16.2	86	15.4
Not getting help	122	4.8	38	4.3	23	4.1
Threat of death	485	19.0	150	16.9	93	16.6
Relocation	51	2.0	14	1.6	7	1.3

*Note*: Pairwise deletion was conducted for missing cases.

^a^ The monthly currency exchange rate in August 2016 was $1.00 = $101.20.

At the 6‐month assessment, 2,554 Japanese residents (*M* age = 47.04 years, *SD* = 12.62; see Table 1) responded. Participants were recruited from the screening study based on having been in any one of the following locations when the earthquake occurred: (a) the primary disaster area, where the Japan Meteorological Agency (JMA) seismic intensity was greater than 6.0 (*M* age = 45.45 years, *SD* = 12.34; *n* = 476 men, *n* = 345 women); (b) the secondary disaster‐damaged area, where JMA seismic intensity was between 4.0 and 5.9 (*M* age = 48.20 years, *SD* = 12.20; *n* = 500 men, *n =* 352 women); or (c) unaffected areas, where JMA seismic intensity was below 4.0 (*M* age = 47.41 years, *SD* = 13.12 years; *n* = 515 men, *n* = 366 women). These participants responded to the other parts of the questionnaire as described in the Measures section. The entire procedure took approximately 30 min or less to complete. Participants were compensated in points from the research company.

Of these participants, we randomly sent 1,200 invitations to participate in the 12‐month assessment. At 12 months, 887 participants responded to the survey (*M* age = 48.11 years, *SD* = 12.43; *n* = 536 men, *n* = 351 women). A total of 560 participants completed measurements at all time points (*M* age = 48.86 years at baseline, *SD* = 12.25). Using the Sidak‐Bonferroni procedure for the number of comparisons for each cross‐tablature, we found that participants who completed the assessments at all time points did not differ from those who did not except that significantly more men completed the study compared to women (24.1% vs. 18.8%, respectively), adjusted standardized residual = 3.2, *p* < .001. In addition, individuals who completed the study were older than those who did not, but the effect size was very small, *t*(2,552) = 3.87, *p* <.001, *d* = 0.16. There were no other differences with regard to the variables listed in Table 1. Because there were no other significant differences in the 6‐month measures used in the following analyses, incomplete cases were treated as missing at random. The current study was approved by the institutional review board at Chuo University.

### Measures

#### PTSS

The 22‐item IES‐R (Weiss & Marmar, 1996), which was used as a screening tool in the present study, is widely used to measure PTSS and includes subscales for symptoms of intrusion, avoidance, and hyperarousal related to trauma exposure. Items are rated on a 5‐point Likert‐type scale ranging from 0 (*not at all*) to 4 (*extremely*), with a total possible score range of 0–88. The scale was originally developed and validated by Horowitz et al. (1979), and the Japanese translation, back‐translation, and validation were conducted by Asukai et al. (1999). In the current study, the IES‐R was used to measure PTSS related to the Tohoku earthquake and tsunami. In the present study, Cronbach's alpha for the total scale was .95.

#### Demographic Characteristics

Sociodemographic characteristics (Table 1) were obtained in the screening study and included age (continuous), gender (dichotomous), subjective marital status (dichotomous), parenthood (dichotomous), highest educational degree (five categories), employment status (six categories), and income level (seven categories).

#### Health‐Related Items

Health‐related items, described by Dougall et al. (2005), such as psychosomatic complaints prior to the disaster (dichotomous), psychosomatic complaints (dichotomous), mental disorders prior to 6 month‐assessment (dichotomous), alcohol consumption (dichotomous), and smoking status (dichotomous) were analyzed for the current study (Table 1). Body mass index and sleep duration were also measured but not used for the current study.

#### Disaster Exposure

Items describing exposure to the earthquake, tsunami, and nuclear crisis, described by used in Dougall et al. (2005), were modified for the current study. Specifically, dichotomous forced‐choice questions (shown in Table 1) were administered. Free‐answer questions, including items related to news exposure, location during the disaster, and descriptions of the disaster experience, were not used in the current study.

#### PTG

The 21‐item Posttraumatic Growth Inventory (PTGI; Tedeschi & Calhoun, 1996) is widely used to measure trauma‐related growth. The PTGI includes five subscales: Relationships with Others (six items), New Possibilities (four items), Personal Strength (four items), Spiritual Change (two items), and Appreciation of Life (three items). Japanese translation and validation were conducted by Taku et al. (2007), who reported that the Spiritual changes and Appreciation of Life subscales were not distinguishable in the Japanese version; thus, the Spiritual changes and Appreciation of Life subscales were combined for the following analyses. Responses are rated using a 6‐point Likert‐type scale ranging from 0 (*did not experience*) to 5 (*to a very great degree*; Taku et al., 2007). The present analyses used PTGI subscale scores from the 6‐, 12‐, and 42‐month assessments. In the present sample Cronbach's alpha values at these time points were .92, .93, and .94, respectively, for the Relationships with Others subscale; .88, .87, and .87 for the New Possibilities subscale; .90, .90, and .92 for the Personal Strength subscale; and .78, .79, and .81 for the combined Spiritual Change/Appreciation of Life subscale. Due to qualitative differences among the subscales (Taku et al., 2007), subscale scores were used for the following analyses.

#### QoL

The Japanese version of the 26‐item World Health Organization (WHO) Quality of Life‐26 (WHOQOL‐26; WHO, 1993) has been widely used to measure QoL, defined as the personal evaluation of one's life condition based on one's own goals, expectations, and standards. The WHOQOL‐26 includes subscales for the domains of physical QoL, psychological QoL, social relationships, and environment. Items are rated on a 5‐point Likert‐type ranging from 1 (*not at all*) to 5 (*extremely*/*completely*). The scale was originally developed and validated by the WHO (1993), and the Japanese translation, back translation, and validation were conducted by Tazaki and Nakane (1997). In the present study, the scale was administered at the 42‐month assessment, and scores were calculated as the mean of the 26 items (range: 1–5). In the present sample, Cronbach's alpha was .93.

### Data Analysis

To explore the number of distinct patterns of change in PTGI total score and subscales and examine the shape of change in the sample, including missing data, group‐based trajectory models (GBTMs) with linear trends (Nagin, 1999) were conducted (*N* = 2,554). Time variables were set as the time elapsed since the 6‐month assessment. We chose to use GBTMs instead of other analysis methods, such as growth mixture modeling (GMM) because within‐trajectory error variance was not the focus of the current study. In addition, trajectory interpretability is more straightforward with GBTM. We entered WHOQOL scores at 42‐month follow‐up as a time‐invariant covariate, resulting in 560 participants in the dataset, which was analogous to listwise deletion. The GBTM was a finite mixture model that estimated regression models for unobserved (i.e., latent) subpopulations based on patterns of change (Van de Schoot, 2015) using the maximum likelihood function. Group membership was assigned to each observation based on posterior probability. This analytic method has become the standard method to estimate unobserved patterns of change for a target population due to its flexibility in dealing with missing observations and various variables (i.e., continuous, count, and dichotomous variables). Regarding the extraction of an adequate number of trajectories, the following criteria were used. First, the Bayes factor represented the exponential difference between the smaller model's Bayesian information criterion (BIC) and the more complex model's BIC (Equation 1) and was used (Nagin, 2005) to select an adequate number of trajectories. If the Bayes factor was smaller than .10, the larger model was supported. In contrast, if the Bayes factor exceeded 10, the smaller model was supported.

(1) BIC.=eBICsmallermodel−BIClargermodel

Finally, the interpretability of trajectories based on theories regarding PTG (Tedeschi & Calhoun, 1996; Zoellner & Maercker, 2006) was used as a criterion to determine the number of trajectories. In each extracted trajectory, linear trends were examined to characterize patterns of change. Based on the criteria, the maximum number of interpretable trajectories were extracted. For the covariate, differences in association (i.e., slope) with WHOQOL scores at 42 months among the trajectories were examined using the Bonferroni procedure, with PTG trajectory as the reference category. When the specification of the reference group changed the trajectory in any sense, we concluded that the trajectories were not stable. Therefore, a more robust, smaller number of trajectories was selected. The associations among trajectory membership and the variables listed in Table 1 and predictors of PTG given in Kyutoku et al. (2012) were explored using neural networks, due to multicollinearity and nonlinear relationships as predictors, in SPSS Modeler (Version 18.2), and none of the variables were estimated to be important (Supplementary Materials). The flow of data collection for the current study is presented in the Supplementary Materials. All preanalysis calculations, such as descriptive statistics and data screening, were performed in SPSS (Version 25.0), and the “proc traj” function (i.e., cnorm with maximum likelihood estimation) in SAS (Version 7.15) was used for the main analyses.

## Results

At the time of the initial screening, the PTSS score (i.e., IES‐R) was 11.95 (*SD* = 12.94, range: 0–61). At each assessment point (Table 1), the largest proportion of participants endorsed the following demographic characteristics: male gender, married, parenthood, and employed full‐time. Many participants earned college degrees. More than 50% of the sample indicated that their income was less than or equal to ¥6,999,999 (JPY; approximately $69,000 USD at the time of measurement) per year. Regarding self‐reported health status, most participants did not report a prior history of psychosomatic complaints or mental disorders. A large number of participants reported alcohol consumption, and most participants were nonsmokers. For disaster experience, a large number of individuals reported having experienced financial loss and/or an acquaintance's suffering due to the disaster. Approximately 1 in 5 participants felt the threat of death. Descriptive statistics for PTG and the PTGI subscales are presented in Table 2. High associations, with pairwise deletion, were found among the PTGI subscales at each time point (i.e., 6‐, 12‐, and 42‐months posttrauma).

**Table 2:** Correlations Among Posttraumatic Growth Inventory (PTGI) Subscales

Variables	1	2	3	4		5	6	7	8	9	10	11	12
6 months													
1. Others	–	.83***	.83***	.83***		.59***	.52***	.52***	.54***	.52***	.45***	.49***	.49***
2. Possibility		–	.84***	.78***		.54***	.55***	.53***	.51***	.47***	.47***	.45***	.45***
3. Strength			–	.78***		.54***	.52***	.61***	.52***	.50***	.47***	.55***	.51***
4. Spirituality/Appreciation				–		.55***	.51***	.52***	.61***	.49***	.46***	.49***	.57***
12 months													
5. Others						–	.86***	.83***	.83***	.54***	.45***	.46***	.52***
6. Possibility							–	.82***	.79***	.47***	.47***	.45***	.47***
7. Strength								–	.78***	.49***	.46***	.51***	.50***
8. Spirituality/Appreciation									–	.47***	.43***	.47***	.55***
42 months													
9. Others										–	.85***	.87***	.84***
10. Possibility											–	.84***	.81***
11. Strength												–	.83***
12. Spirituality/Appreciation													–
*N*	2554		887	560									
*M*_sum_	7.64	4.33	4.61	4.77		8.03	4.54	2.98	5.11	7.11	4.09	4.46	4.69
*SD*	6.57	4.30	4.53	4.12		6.51	4.20	4.55	4.20	6.58	4.16	4.64	4.29

*Note*. Pairwise deletion was used for missing values among time points. Others = Relating to Others subscale; Possibility = New Possibilities subscale; Strength = Personal Strength subscale; Spiritual/Appreciation = Spiritual Change/Appreciation of Life subscales.

*** *p* < .001.

### PTGI Subscale Trajectories and Sample Proportions

The proportions of participants who were categorized in each PTGI subscale trajectory group are herein described (Table 3).

**Table 3:** Criterion‐Related Estimates for Posttraumatic Growth Inventory (PTGI) Total Score and Subscale Trajectories

Subscale and number of trajectories	Trajectory size (% of sample)					LL	AIC	BIC	Bayes factor (smaller–larger)
Others									
2	64.3	34.8				−5,284.5	−5,291.5	−5,306.7	< 0.1
3	53.1	34.4	12.5			−5,241.0	−5,252.0	−5,275.8	< 0.1
4	49.2	20.7	15.5	14.6		−5,219.3	−5,234.3	−5,266.7	> 10
5	44.1	21.1	14.6	14.1	6.2	−5,214.8	−5,233.8	−5,274.9	
Possibility									
2	73.0	27.0				−4,570.6	−4,577.6	−4,592.8	< 0.1
3	65.5	29.9	4.7			−4,538.1	−4,549.1	−4,572.9	< 0.1
4	56.9	18.0	14.6	10.4		−4,511.3	−4,526.3	−4,558.7	< 0.1
5	52.7	16.9	16.1	11.4	2.9	−4,484.3	−4,503.3	−4,544.4	
Strength									
2	69.7	30.3				−4,649.1	−4,656.1	−4,671.2	< 0.1
3	60.6	30.6	8.7			−4,603.4	−4,614.4	−4,638.2	> 10
4	60.7	30.6	8.7	0.0		−4,603.4	−4,618.4	−4,650.8	< 0.1
5	50.8	18.4	13.1	12.1	5.7	−4,563.0	−4,582.0	−4,623.2	
Spirituality/Appreciation									
2	67.4	32.6				−4,541.5	−4,548.5	−4,563.7	< 0.1
3	55.9	35.7	8.4			−4,476.1	−4,487.1	−4,511.0	< 0.1
4	54.7	25.0	13.5	6.8		−4,454.3	−4,469.3	−4,501.7	< 0.4
5	47.2	20.6	17.8	10.8	3.7	−4,440.8	−4,459.8	−4,500.9	

*Note*. LL = log‐likelihood; AIC = Akaike information criterion; BIC = Bayesian information criterion; Others = Relating to Others subscale; Possibility = New Possibilities subscale; Strength = Personal Strength subscale; Spiritual/Appreciation = Spiritual Change/Appreciation of Life subscales.

#### PTGI Relationships With Others Subscale Trajectories

**Figure 1:**
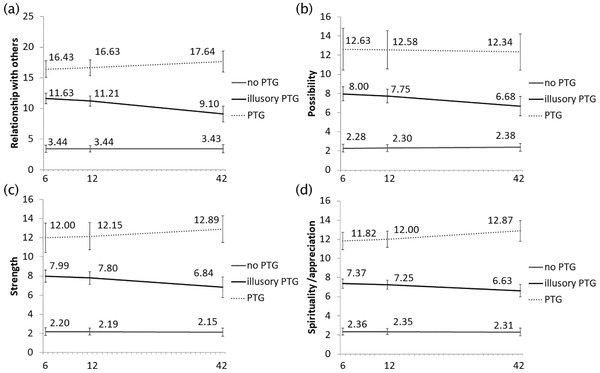
Posttraumatic Growth (PTG) Trajectories for the (A) Relationships with Others, (B) New Possibilities, (C) Personal Strength, and (D) Spiritual Change/Appreciation of Life Subscales of the PTG Inventory

Based on Bayes factors, four trajectories appeared to be appropriate for the PTGI Relationships with Others subscale (Fig. 1, Panel A; Table 3). However, specifying the PTG trajectory as the reference group slightly changed the trajectory patterns due to differences in the iterative process, indicating an unstable solution. Therefore, a three‐trajectory solution, which is consistent with previous findings, was used for the subsequent analyses. The first trajectory was named “no PTG,” as it showed a consistently very low degree of PTG (no linear trend), *p* = .990. The second trajectory was named “illusory PTG” because of a linearly decreasing trend, *p* < .001. The third trajectory was named “PTG” because we regarded the consistently high degree of PTG (i.e., no linear trend), *p* = .167, as a stable transformational positive change. As expected, compared with members in the PTG trajectory group, members in the no PTG and illusory PTG groups showed a more negative slope, −1.652 (*SE* = .322), *p* < .001, and −1.264 (*SE* = .342), *p* < .001, respectively, for WHOQOL scores at 42 months (see Table 4 for WHOQOL‐26 mean scores and standard errors).

**Table 4:** World Health Organization Quality of Life–26 (WHOQOL‐26) Scores at 42‐Month Follow‐Up, by Trajectories

	*WHOQOL‐26* score, by PTG trajectory											
	No PTG				Illusory PTG				PTG			
PTGI subscale	*M*	*SE*	*n*	*%*	*M*	*SE*	*n*	*%*	*M*	*SE*	*n*	*%*
Others	3.09	0.03	299	53.4	3.20	0.04	195	34.8	3.61	0.06	66	11.9
Possibility	3.10	0.03	373	66.6	3.31	0.04	165	29.5	3.66	0.10	22	3.9
Strength	3.07	0.03	340	60.7	3.28	0.04	179	32.0	3.75	0.09	41	7.3
Spirituality/Appreciation	3.07	0.03	311	55.5	3.19	0.04	62	11.1	3.50	0.08	38	6.9
Multigroup trajectory	3.06	0.04	256	45.7	3.20	0.04	179	32.0	3.46	0.05	125	22.3

*Note. N* = 560.

#### PTGI New Possibilities Subscale Trajectories

Based on the criteria, none of the models satisfied the Bayes factor for the New Possibilities subscale. Nagin (2005) suggests the extraction of the maximum number of meaningful trajectories based on previous findings, but a five‐trajectory model showed redundant trajectories. Therefore, four trajectories appeared to be appropriate for the New Possibilities subscale (Figure 1, Panel B; Table 3). However, specifying the PTG trajectory as the reference group slightly changed the trajectory patterns due to differences in the iterative process, indicating an unstable solution. Therefore, a three‐trajectory solution, which is consistent with previous findings, was used for the subsequent analyses. The first trajectory was named no PTG, as it consistently showed a very low degree of PTG (i.e., no linear trend), *p* = .672. The second trajectory was named illusory PTG because of the linearly decreasing trend, *p* = .002. The third trajectory was named PTG, as consistently high of PTG emerged (i.e., no linear trend), *p* = .785, indicating a stable, transformational positive change. Compared with members in the PTG trajectory, members in the no PTG and illusory PTG trajectories showed a more negative slope, ‐1.779 (*SE* = .468), *p* < .001, and ‐1.157 (*SE* = .485), *p* < .034, respectively, for WHOQOL scores at 42‐months posttrauma, as expected (see Table 4 for WHOQOL‐26 mean scores and standard errors).

#### PTGI Personal Strength Subscale Trajectories

Based on the criteria and interpretability of the previous findings, three trajectories appeared to be appropriate for the Personal Strength subscale, but they varied slightly (Figure 1, Panel C; Table 3). The first trajectory was categorized as no PTG based on a consistently low degree of PTG (no linear trend), *p* = .844. The second trajectory was classified as illusory PTG due to a linearly decreasing trend, *p* = .011. The third trajectory was named PTG based on the consistently high degree of PTG that emerged (no linear trend), *p* = .217. Members in the no PTG, slope = −2.122 (*SE* = .424), *p* < .001, and illusory PTG trajectories, slope = −1.478 (*SE* = .420), *p* < .001, showed a more negative slope compared to those in the PTG trajectory with respect to WHOQOL scores at 42‐months posttrauma, as expected (see Table 4 for WHOQOL‐26 mean scores and standard errors).

#### PTGI Spiritual Change/Appreciation of Life Subscale Trajectories

Based on the criteria, none of the models satisfied the Bayes factor. Based on recommendations by Nagin (2005), we extracted the maximum number of meaningful trajectories based on previous findings; however, a five‐trajectory model showed redundant trajectories. Therefore, four trajectories appeared to be appropriate for the Spiritual Change/Appreciation of Life subscale (Figure 1, Panel D; Table 3). However, specifying the PTG trajectory as the reference group slightly changed the trajectory patterns, indicating an unstable solution due to differences in the iterative process. Therefore, consistent with previous findings, a three‐trajectory solution was used for the subsequent analyses. The first trajectory was categorized by no PTG, as it showed a consistently very low degree of PTG (no linear trend), *p* = .672. The second trajectory was categorized by illusory PTG, as a linearly decreasing trend emerged, *p* = .002. The third trajectory was categorized by PTG, as we regarded the consistently high degree of PTG (no linear trend), *p* = .785, to represent a stable, transformational positive change. Members in the no PTG trajectory (slope = −1.423, *SE* = .340, *p* < .001) showed a lower slope for WHOQOL scores at 42‐months posttrauma compared with those in the PTG trajectory but not those in the illusory PTG trajectory (slope = −0.660, *SE* = 0.358, *p* = .130; see Table 4 for WHOQOL‐26 mean scores and standard errors).

### Exploration of Homogeneity and Heterogeneity Among PTGI Subscales

There was heterogeneity among PTGI subscales (Table 4). Based on the individual subscales’ trajectories, multigroup analysis, which allows for simultaneous estimation of trajectories for four subscales (Nagin et al., 2016), was conducted by specifying three trajectories, with QoL entered as a risk factor. The results showed that the heterogeneity between individual and multigroup analyses indicated that constraining the trajectories across subscales was not appropriate (Table 4). As the descriptive statistics indicate, there was heterogeneity among trajectories. The coincidence rates for trajectories between any pair of PTGI subscales were moderately high, as shown in Table 5. However, the coincidence rates of the illusory PTG and PTG trajectories across all subscales were low (i.e., .29 and .15, respectively), whereas the no PTG trajectory showed moderate coincidence (i.e., .66). These results indicate that individuals’ trajectory patterns were not uniform across all PTGI subscales. Thus, despite the high correlation with the PTGI total score (Table 2), each subscale tended to show a distinct pattern of trajectories. We further explored heterogeneity of trajectory membership across PTGI subscales as well as inconsistent patterns within each trajectory (see Supplementary Materials).

**Table 5:** Coincidence Rate of Trajectories Among Posttraumatic Growth Inventory Subscales

Subscale	1	2	3	4
1. Others	–	.77	.80	.80
2. Possibility		–	.82	.81
3. Strength			–	.82
4. Spiritual/Appreciation				–

*Note*. Others = Relating to Others subscale; Possibility = New Possibilities subscale; Strength = Personal Strength subscale; Spiritual/Appreciation = Spiritual Change/Appreciation of Life subscales.

## Discussion

The current findings revealed a pattern of PTG change 6‐, 12‐, and 42‐month assessment points following the 2011 Tohoku earthquake and tsunami and subsequent Fukushima I nuclear power plant disaster, mostly supporting the existence of distinct PTG trajectories. In addition, in partial support of our hypotheses, we found that PTG trajectory membership was associated with QoL in different ways. As expected, participants in the PTGI subscale trajectories categorized by PTG reported higher levels of QoL compared with those in the no PTG or illusory PTG trajectories. These findings indicate the importance of conducting longitudinal studies to elucidate the diversity in the ways PTG can change.

Overall, three trajectory patterns were extracted for PTGI subscales: no PTG, illusory PTG, and PTG. These trajectories are analogous to previously reported PTSS trajectory patterns (Johannesson et al., 2015; Magruder et al., 2016), except that our results did not support the late‐onset PTG trajectory found in previous work. For all PTGI subscales, most participants were categorized into the no PTG trajectory. A certain proportion of individuals appeared to cope with early‐phase PTE exposure by perceiving illusory growth, which begins to decrease around one‐year posttrauma. Because of the relatively low rate of emergence, the effect caused by this population was overlooked without decomposing the trajectories. A PTG trajectory was observed for all subscales.

Regarding the second study objective, members of the PTG trajectory groups generally reported higher levels of QoL compared with those in the no PTG and illusory PTG trajectories, thus supporting our second hypothesis. This indicates the validity of the PTG trajectories. Thus, in contrast to findings reported by Bonnano (2005), most individuals in the no PTG trajectory group did not seem to be resilient. The current finding that membership in the illusory PTG group was not associated with higher QoL scores supports the idea that illusory PTG is a temporal, superficial coping mechanism that is not salubrious, as suggested by Zoellner and Maercker's (2006) idea of the illusory faces of PTG. The association between PTG trajectories and QoL indicates that there was both actual growth, which enhanced positive change later, and pseudogrowth, which was not associated with enhanced QoL. Thus, it is important to note that early detectable growth could actually have been related to coping and not necessarily associated with actual growth. In addition, different patterns of association between PTG and QoL reported in previous findings (Bellizzi et al., 2010; Linley & Joseph, 2004; Teodorescu et al., 2012) may be explained by different types of PTG trajectories. There may have been a time lag reflecting QoL growth depending on the facet of PTG. Hence, members of the PTG trajectory group appeared to experience the benefits of actual growth.

Despite the high correlations among subscales, coincidence rates between trajectory membership across all subscales were not homogeneous. This emphasizes the importance of probing PTG trajectories at the subscale level to more efficiently intervene with regard to specific aspects of growth to produce beneficial transformative change that can lead to improved QoL. Regarding correlates of trajectories in each PTGI subscale, none of the variables listed in Table 1 and nor the predictors of PTG, based on our previous findings (Kyutoku et al., 2012), sufficiently predicted trajectory membership. Thus, the course of PTG may have been a facet‐specific process. We recommend that future researchers explore PTGI subscales when examining PTG trajectories.

Despite numerous study strengths, some limitations must be discussed. There was a high attrition rate due to the nature of the disaster and the longitudinal study design. For example, there were more completed cases among men than women. Further, data obtained at 12‐months postdisaster was limited due to financial constraints. This might have attenuated the generalizability of the current findings. In addition, although the PTG trajectories were consistent with previous findings, only the Personal Strength subscale met the statistical criteria, trajectory size, and interpretability standards at once. Therefore, the generalizability of the rest of the PTGI subscales may be attenuated. The PTG construct may have been measured differently in the Japanese version of the PTGI compared to the original English version (Taku et al., 2008; Tedeschi & Calhoun, 1996). Consequently, caution should be used in generalizing the current findings to other cultures. Further, a growth‐mixture model that utilized within‐trajectory variance (Muthen, 2001) and a joint‐trajectory model that extracted common trajectories between two types of measurements (e.g., Dugre et al., 2019) could have been utilized had they been relevant to the research aims. Finally, because participants were classified into one trajectory using GBTM (Nagin, 2005), those who showed a low probability (Mori et al. 2020) of trajectory assignment for the PTGI Relationships With Others subscale are examined in the Supplementary Materials.

The current findings contribute to the theoretical understanding of PTG, with implications for developing efficient PTG‐related interventions to improve QoL. To the best of our knowledge, this was the first study to emphasize the importance of PTGI subscale trajectories regarding QoL using a group‐based trajectory model that incorporated a longitudinal design. We believe that the current findings suggest that each model of PTG partially reflects its nature.

## Open Practices Statement

The current study reported in the current article was not formally preregistered. The data have not been made available on the permanent third‐party archive, as the institutional review board ruled that we were not permitted to post the data. Questions regarding data should be sent to the lead author at qworkdog@gmain.com .

## Supporting information

Supporting Material
