# Supplementary material for: Trajectories of Posttraumatic Growth and Their Associations With Quality of Life After the 2011 Tohoku Earthquake and Tsunami
Source: J Trauma Stress. 2020 Nov 23;34(3):512–25. doi: 10.1002/jts.22628 (PMC8246896; doi:10.1002/jts.22628)
Supplement: Supplementary file 1 — Supporting Material [file JTS-34-512-s001.docx]

**Supplementary materials**

**Suppl. 1 Exploration of correlated variables with PTG trajectories**

**Data analyses**

The associations among variables in Table 1, the predictors of PTG used in Kyutoku et al. (2012), and the PTG trajectories for each PTG subscale were explored using multilayer perceptron (MLP). MLP is a type of neural network model with multiple perceptron and layers, which is composed of an input layer (predictors), hidden layer(s), and output layer.

The input layer comprises the initial neurons, which receive unprocessed raw data. Each neuron in the input layer thereby is responsible for handling one input variable, which it transforms via the activation function and then passes the outcome onto the next layer neurons. (Wendler & Gurottrup, 2016)

By these outcomes, the output neurons classify the category of target variable for a classification task (e.g. Suppl. Fig. 1, 2, 3, and 4). The hyperbolic tangent function has been commonly used as an activation function for the hidden layer to efficiently conduct the analyses, and the softmax function was used as an activation function for the output layer. MLP enables the classification of complex boundaries, which is useful for exploratory purposes. Therefore, MLP was used to account for dependent variables with complex borders like PTG trajectory. The original data were randomly assigned to learning (60%), validation (20%) and test (20%) data to avoid overfitting. Classification accuracy for each data set, overall classification table and importance index, which was a standardized relative importance of predictors for classification, exceeding .10 in the test data were interpreted. Predictors with high importance indices were indicated by thick nodes in Suppl. Fig. 1, 2, 3, and 4. SPSS Modeler 18.2 was used for the analyses.

**Results**

**Prediction of each PTGI subscale trajectory using variables in Table 1 and predictors of PTG in Kyutoku et al. (2012) as predictors**

**PTGI others.** Variables in Table 1 and predictors of PTG used in Kyutoku et al. (2012) were used for the classification of no PTG, illusory PTG and PTG trajectories. The MLP model was composed with an input layer, a hidden layer with three neurons, and an output layer (Suppl. Fig. 1). Although PTSS at screening (.21) and challenge at screening (.25) showed moderate importance, the results indicated that classification accuracy was low: 49.6%. Accuracy for validation and test data were 46.6% and 46.4%, respectively. According to the classification table (Suppl. Table 1), a substantial proportion of members in the illusory PTG trajectory and the PTG trajectory were misclassified (correct classification = .64).

Suppl. 1 Table 1

Classification table for PTG others trajectories in percentage for overall data

| Observed |  | Predicted |  |
| --- | --- | --- | --- |
|  | No PTG | Illusory PTG | PTG |
| No PTG | **85.5%** | 11.8% | 2.6% |
| Illusory PTG | 50.5% | **45.5%** | 4.0% |
| PTG | 27.8% | 47.2% | **25.0%** |

*Note*. Coincidence rate is shown in bold on the diagonal.

Suppl. 1 Fig. 1 MLP to classify PTG relationship with others trajectories

Notes. Thickness of nodes corresponds to importance of predictors.

**PTGI new possibility.** Variables in Table 1 and predictors of PTG used in Kyutoku et al. (2012) were used for the classification of no PTG, illusory PTG and PTG trajectories. The MLP model was composed with an input layer, a hidden layer with a neuron, and an output layer (Suppl. Fig. 2). Although PTSS at screening (.20) and challenge appraisal at screening (.27) showed moderate importance, the results indicated that classification accuracy was low: 50.6%. Accuracy for validation and test data were 47.3% and 48.0%, respectively. According to the classification table (Suppl. Table 2), a substantial proportion of members in the illusory PTG trajectory and the PTG trajectory were misclassified (correct classification = .71).

Suppl. 1 Table 2

Classification table for PTG new possibility trajectories in percentage for overall data

| Observed |  | Predicted |  |
| --- | --- | --- | --- |
|  | No PTG | Illusory PTG | PTG |
| No PTG | **82.5%** | 17.5% | 0.0% |
| Illusory PTG | 44.6% | **55.4%** | 0.0% |
| PTG | 25.0% | 75.0% | **0.0%** |

*Note*. Coincidence rate is shown in bold on the diagonal.

Suppl. 1 Fig. 2 MLP to classify PTG new possibility trajectories

Notes. Thickness of nodes corresponds to importance of predictors.

**PTGI strength.** Variables in Table 1 and predictors of PTG used in Kyutoku et al. (2012) were used for the classification of no PTG, illusory PTG and PTG trajectories. The MLP model was composed with an input layer, a hidden layer with two neurons, and an output layer (Suppl. Fig. 3). Although PTSS at screening (.18) and challenge appraisal at screening (.27) showed moderate importance, the results indicated that classification accuracy was low: 50.7%. Accuracy for validation and test data were 47.5% and 46.4%, respectively. According to the classification table (Suppl. Table 3), a substantial proportion of members in the illusory PTG trajectory and the PTG trajectory were misclassified (correct classification = .73).

Suppl. 1 Table 3

Classification table for PTG strength trajectories in percentage for overall data

| Observed |  | Predicted |  |
| --- | --- | --- | --- |
|  | No PTG | Illusory PTG | PTG |
| No PTG | **93.7%** | 6.3% | 0.0% |
| Illusory PTG | 57.6% | **38.0%** | 4.3% |
| PTG | 13.0% | 34.8% | **52.2%** |

*Note*. Coincidence rate is shown in bold on the diagonal.

Suppl. 1 Fig. 3 MLP to classify PTG strength trajectories

Notes. Thickness of nodes corresponds to importance of predictors.

**PTGI spirituality.** Variables in Table 1 and predictors of PTG used in Kyutoku et al. (2012) were used for the classification of no PTG, illusory PTG and PTG trajectories. The MLP model was composed with an input layer, a hidden layer with one neuron, and an output layer (Suppl. Fig. 4). Although PTSS at screening (.19) and challenge appraisal at screening (.22) showed moderate importance, the results indicate that classification accuracy was low: 50.1%. Accuracy for validation and test data were 47.1%, and 46.3%, respectively. According to the classification table, a substantial proportion of members in the illusory PTG trajectory and PTG trajectory were misclassified (correct classification = .68).

Supple. 1. Table 4

Classification table for PTG spirituality trajectories in percentage for overall data

| Observed |  | Predicted |  |
| --- | --- | --- | --- |
|  | No PTG | Illusory PTG | PTG |
| No PTG | **87.9%** | 12.1% | 0.0% |
| Illusory PTG | 48.5% | **51.5%** | 0.0% |
| PTG | 7.4% | 92.6% | **0.0%** |

*Note*. Coincidence rate is shown in bold on the diagonal

Suppl. 1 Fig. 4 MLP to classify PTG spirituality trajectories

Notes. Thickness of nodes corresponds to importance of predictors.

**Conclusion**

The variables in Table 1 and predictors of PTG in our previous findings (Kyutoku et al., 2012) were used as predictors to classify the trajectories of PTGI subscales using MLP. The results indicated that classification accuracy was low for each subscale. It seemed very difficult to predict PTG trajectory during the relatively initial phase of post-disaster psychological adjustment. That implied that demographic, past health history, disaster experience, and psychological constructs (Table 1 and Kyutoku et al., 2012) were not sufficient predictors to classify trajectory membership in advance. Thus, trajectory of PTG would be rather unpredictable by demographics, health status, disaster experience, and psychological constructs that were found to be correlates of PTG including PTSS and cognitive appraisal at screening (see descriptive statistics in Kyutoku et al. 2012).

**References**

Kyutoku, Y., Tada, R., Umeyama, T., Harada, K., Kikuchi, S., Watanabe, E., Liegey-Dougall, A. & Dan, I. (2012). Cognitive and psychological reactions of the general population three months after the 2011 Tohoku earthquake and tsunami. *PLoS ONE, 7*(2): e31014. https://doi.org/10.1371/journal.pone.0031014

Wendler, T., & Grottrup, S. (2016). Data Mining with SPSS Modeler (pp.845). Springer.

**Suppl. 2 Flow of the current study**

Suppl. 2 Fig. 1 Flowchart of the current study

**Suppl. 3 Heterogeneity of trajectory membership across subscales**

In addition to multi-group analyses, trajectories in a pair of others and possibility subscales were explored using a joint trajectory model. The others subscale was used as the first model and the new possibility subscale was used as the second model. As for the staring value, parameter estimates were based on the parameter estimates for the individual model with QoL as a time invariant covariate. This allowed simultaneous estimation of trajectories for both subscales. Also, other pairs of joint trajectory models were briefly disused. Detailed explanation about the analysis was presented in Jones & Nagin, 2007, and an example of research application was reported in Dugre et al, 2019.

**Result & Discussion**

Trajectories for others and possibility subscales were simultaneously estimated using parameters of each subscale from the individual GBTM (Suppl. 3 Fig. 1). For the others subscale, the following linear trends were observed: no PTG (no linear trend, *p* = .11), illusory PTG (decreasing linearly trend, *p* < .001), and PTG (no linear trend, *p* = .20). As for the group membership, 52.7% were categorized as no PTG trajectory (*p* < .001), 29.2% as illusory PTG trajectory (*p* < .001) and 18.1 as PTG trajectory (*p* < .001). For the possibility subscale, the following linear trends were observed: no PTG (slightly increasing linear trend, *p* = .008; 52.6%), illusory PTG (decreasing linear trend, *p* < .001; 29.8%), and increasing PTG (linear trend, *p* = .004; 17.6%). As for the group membership given the others subscale parameters, 99.9% of the no PTG trajectory in the others model were categorized as no PTG trajectory in the possibility subscale. As for agreements, all in the illusory PTG trajectory were categorized as illusory PTG trajectory, and 96.9% of the PTG trajectory in the others subscale were categorized as PTG trajectory. About three percent of the PTG trajectory of the others subscale were categorized as illusory PTG. Overall, 53.1% were categorized to no PTG trajectory, 34.4% to illusory PTG trajectory, and 12.5% to PTG trajectory. Results from reversing others as model 2 and possibility subscale as model 1were akin to these findings. Additionally, we briefly discuss the examination of all pairs of joint trajectory models, specifically others-strength, others-spirituality, possibility-strength, and strength-spirituality. The reversed orders of joint trajectory models were not examined for these analyses. The agreement of trajectories for any pair of subscales was high. Strength given others (lowest agreement = .93 in PTG trajectory), spirituality given strength (lowest agreement = .92 in PTG trajectory), strength given possibility (lowest agreement = .94 in PTG trajectory), and possibility given spirituality (lowest agreement = .92 in PTG trajectory), and strength given spirituality (lowest agreement = .93 in PTG trajectory) showed below 95% agreement, indicating that PTG trajectory in strength subscale appeared to have a somewhat distinct trajectory membership across subscales. As for the shape of the trajectory, no PTG showed increasing linear trend (*p* < .05) and PTG showed decreasing linear trend (*p* < .05) in strength given others, strength given possibility, and spirituality given possibility as opposed to the individual analyses. As opposed to the individual analyses, no PTG showed increasing linear trend (*p* < .05) in strength subscale given others subscale. As opposed to individual analysis, PTG showed linearly decreasing trend (*p* < .05) in spirituality given strength. Thus, the trend of extracted trajectory and proportion of membership differ among individual trajectory, multi-trajectory model, and joint trajectory models (Table 4, Fig. 1, Suppl. 3. Fig. 1). When existence of heterogeneity among subscales (i.e. not consistent trajectory membership across subscales or inconsistent trajectory patterns) are known, the appropriate trajectory model should be used to be in line with the research purpose. For instance, the individual examination of a subscale would be appropriate when tailored intervention for specific aspects of growth is intended while a multi-/joint-trajectory model would be appropriate when more integrative insights from the analyses are desired.

**Suppl. 3 Fig. 1 Joint trajectory of PTG others (a) and Possibility (b) subscales**

*Note.* The values in the table represent the mean scores at each time point and error bars represent 95% confidence intervals.

**References**

Dugre, J. R., Dumais, A., Dellazizzo, L, & Potvin S. (2019). Developmental joint

trajectories of anxiety-depressive trait and trait-aggression: implications for co-occurrence of internalizing and externalizing problems. *Psychological Medicine, 50*(8), 1338-1347. doi: 10.1017/S0033291719001272

Jones, B. L. & Nagin, D. S. (2007). Advances in Group-Based Trajectory Modeling and

an SAS Procedure for Estimating Them. *Sociological Methods & Research,35*(4), 542-572. [https://doi.org/10.1177/0049124106292364](https://doi.org/10.1177%2F0049124106292364)

**Suppl. 4 Heterogeneity within a trajectory**

Heterogeneity within a solution was explored. Participants who showed poor class assignments (p < .70; Mori et al., 2020) were examined for each subscale to elucidate the pattern of mal-classification. A one-way between-subjects MANOVA was conducted to examine the systematic difference in the variables in GBTM (PTG time1-time3 and WHOQOL) between participants who showed sufficient fit and poor fit. If there was no distinct pattern of differences, individual data were discussed by line graph reflecting the time course of PTG.

**Results & Discussion**

**Misclassification in others subscale**

Seventy participants (12.5%) were poorly classified. For the no PTG trajectory, 20 participants were poorly classified. Ratings of variables used in GBTM for those who showed poor fit were significantly higher, *F*(4, 294) = 19.281, *p* < .001, η^2^ = .202 (Suppl. 4 Table 1). Individual time course of poorly classified participants were shown in Suppl. 4. Fig. 1.

For the illusory PTG trajectory, 36 participants (18.4%) were poorly classified. Ratings of variables used in GBTM did not significantly differ between participants who showed good fit and poor fit, *F*(4, 190) = 2.21, *p* = .069, η^2^ = .044, indicating that there was not systematic differences (Suppl. 4 Table 1). As for individual time course, the line plot (Suppl.4 Fig. 2) indicated that they did not show linearly decreasing patterns, which characterized the illusory trajectory, during the measurements.

As for the PTG trajectory, 14 (21.2%) of participants were poorly classified. Ratings of variables used in GBTM for those who showed poor fit were significantly lower, *F*(4, 51) = 5.977, *p* < .001, η^2^ = .282, indicating that PTG ratings of those who showed poor fit were consistently lower (Suppl. 4 Table 3). Individual time courses of poorly classified participants were shown in Suppl. 4. Fig. 3.

Thus, both individual pattern and systematic pattern were associated with poor classification in the current data. Caution should be used in dealing with these participants. The probability of each individual’s classification, or weighted value depending on certainty of classification, would be used for future studies to take heterogeneity within a trajectory into consideration.

Suppl. 4 Table 1

Difference between those who showed sufficient and poor fits in no PTG trajectory

Suppl. 4 Table 2

Difference between those who showed sufficient and poor fits in illusory PTG trajectory

Suppl. 4 Table 3

Difference between those who showed sufficient and poor fits in PTG trajectory

**Suppl. 4 Fig. 1 Individual time course of members in lo PTG trajectory**

**Suppl. 4 Fig. 2 Individual time course of members in illusory PTG trajectory**

**Suppl. 4 Fig. 3 Individual time course of members in PTG trajectory**

**Reference**

Mori, M., Krumholz, H. M., & Allore, H. G. (2020). Using Latent Class Analysis to

Identify Hidden Clinical Phenotypes. *Clinical Review & Educaton, 324*(7), 700-701. doi:10.1001/jama.2020.2278
